# Supplementary material for: S/O/W Emulsion with CAPE Ameliorates DSS-Induced Colitis by Regulating NF-κB Pathway, Gut Microbiota and Fecal Metabolome in C57BL/6 Mice
Source: Nutrients. 2024 Apr 12;16(8):1145. doi: 10.3390/nu16081145 (PMC11054280; doi:10.3390/nu16081145)
Supplement: Supplementary file 1 [file nutrients-16-01145-s001.zip › nutrients-2939537-supplementary.pdf]

Supplementary Figure S1

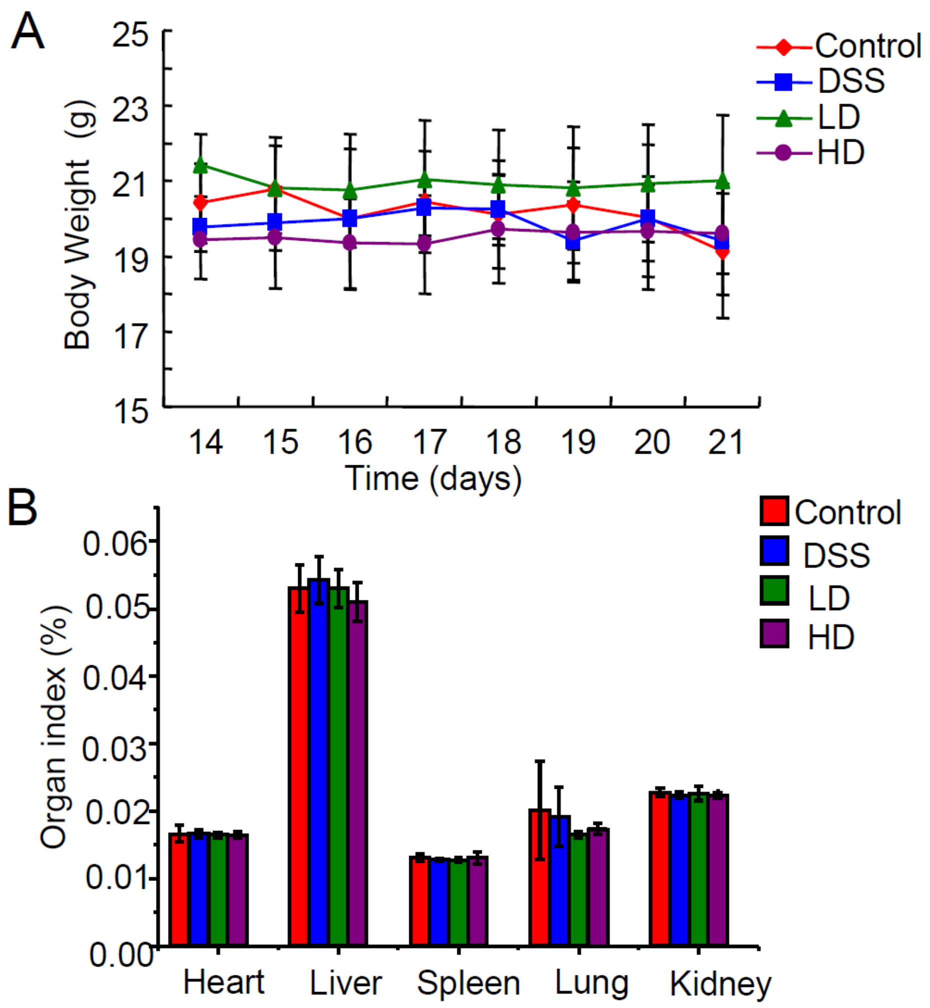

Figure S1 Effect of the CAPE-emulsion on the body weight (A) and organ indices (B) of DSS-induced colitis mice.

Supplementary Figure S2

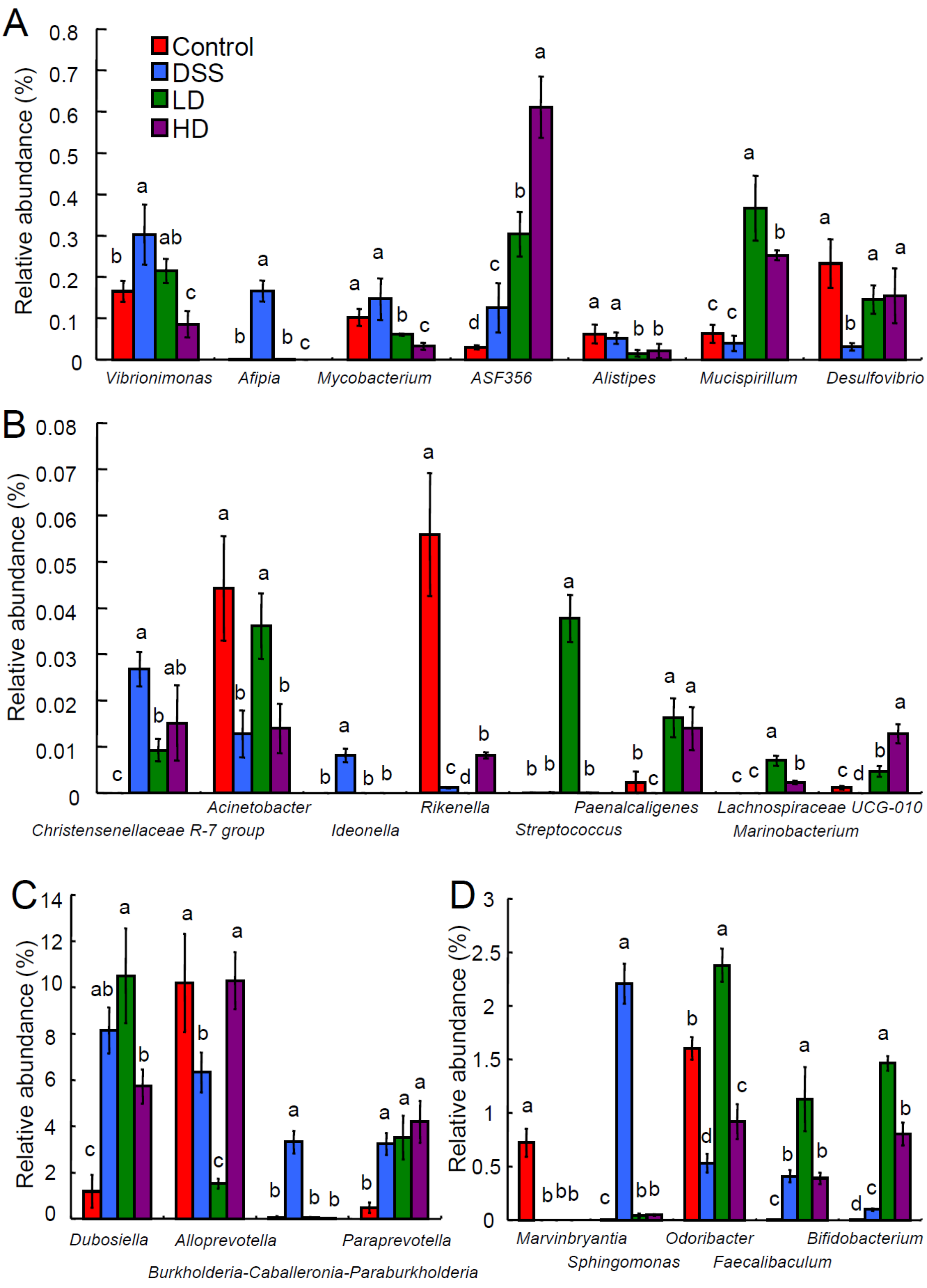

Figure S2 Effect of the CAPE-emulsion on the relative abundance of the biomarkers by LEfSe analysis in DSS-induced colitis mice. The data are expressed as the mean ± SEM with different letters in the same genus indicating significant differences (P< 0.05, n = 3).
